# Twenty New Records of Bees (Hymenoptera, Apoidea) for Sardinia (Italy)

**DOI:** 10.3390/insects12070627

**Published:** 2021-07-10

**Authors:** Vittorio Nobile, Roberto Catania, Pietro Niolu, Michelina Pusceddu, Alberto Satta, Ignazio Floris, Simone Flaminio, Salvatore Bella, Marino Quaranta

**Affiliations:** 1 Indipendent Researcher, 97100 Ragusa, Italy; nobilevittorio@tin.it; 2 Centro di Ricerca Olivicoltura, Frutticoltura e Agrumicoltura, (CREA) Consiglio per la Ricerca in Agricoltura e l’analisi dell’Economia Agraria, Corso Savoia 190, 95024 Acireale, Italy; robertocatania1995@libero.it; 3 Indipendent Researcher, 07041 Alghero, Italy; pietroniolu@libero.it; 4 Department of Agricultural Sciences, University of Sassari, Viale Italia 39, 07100 Sassari, Italy; mpusceddu@uniss.it (M.P.); albsatta@uniss.it (A.S.); 5 Centro di Ricerca Agricoltura e Ambiente, (CREA) Consiglio per la Ricerca in Agricoltura e l’analisi dell’Economia Agraria-via di Corticella 133, I-40128 Bologna, Italy; simone.flaminio@crea.gov.it (S.F.); marino.quaranta@crea.gov.it (M.Q.)

**Keywords:** Colletidae, Andrenidae, Halictidae, Megachilidae, Apidae, Mediterranean, pollinators, wild bees, Sardinia, biogeography, distribution, fauna

## Abstract

**Simple Summary:**

Recently, several studies have highlighted the global decline of pollinators. This has increased concern for the long-term sustainability of plant biodiversity, food production, human nutrition, and human well-being. In Europe, approximately 40% of bee species are threatened in some countries. The Mediterranean Basin is one of the richest areas of diversity of wild bees. Sardinia, the second largest island in the basin, has a flora and fauna with many endemic taxa. Until recently, a number of 316 species of bees was reported on the island. However, the identification of the bee fauna of Sardinia still remains incomplete compared to other islands of the Mediterranean Basin, such as Sicily, Malta, Cyprus, and the Balearic Islands. It is very important to know and protect the potentially rich bee biodiversity of Sardinia, considering that the different factors that now threaten wild pollinators in Europe could lead to its impairment. This paper reports 20 new records of bee species from Sardinia based on observations made in different coastal and mountain ecosystems of the island. These findings contribute to the knowledge of the systematics and distribution of the Sardinian bee fauna.

**Abstract:**

In Sardinia, the second largest Mediterranean island, 316 species of bees are known. Here, for the first time, the following 20 *taxa* are reported: *Colletes cunicularius* (Linnaeus, 1761), and *C. eous* Morice, 1904 (Colletidae); *Andrena humilis* Imhoff, 1832, *A. granulosa* Pérez, 1902, *A. cineraria* (Linnaeus, 1758), *A*. *pallitarsis* Pérez, 1903, *A. rugulosa* Stöckhert, 1935, *A. savignyi* Spinola, 1838, and *A. tenuistriata* Pérez, 1895 (Andrenidae); *Sphecodes reticulatus* Thomson, 1870 (Halictidae); *Lithurgus tibialis* Morawitz, 1875, *Chelostoma emarginatum* (Nylander, 1856), *Dioxys cinctus* (Jurine, 1807), *Coelioxys caudatus* Spinola, 1838, *C. obtusus* Pérez, 1884, and *Megachile ericetorum* (Lepeletier, 1841) (Megachilidae); and *Nomada melathoracica* Imhoff, 1834, *N. pulchra* Arnold, 1888, *Eucera proxima* Morawitz, 1875 and *Tetralonia malvae* (Rossi, 1790) (Apidae). *N. pulchra* is reported for the first time in Italy.

## 1. Introduction

One of the major hotspots for plant and animal diversity in Europe and the world is the Mediterranean Basin, which is characterized by a high incidence of endemic entities (species or subspecies) [1] and is one of the richest areas of diversity of wild bees (Hymenoptera, Apoidea) [2,3,4].

Recently, several reviews have highlighted the global decline of insects [5,6] including pollinators [7,8,9]. This has increased concern for the long-term sustainability of plant biodiversity, food production, human nutrition, and human well-being. The International Union for Conservation of Nature and Natural Resources (IUCN) European Red List for bees shows that 37% of bee species suffer from declining populations. Approximately 9% of all bees and 26% of bumblebees are classified as threatened, whereas 57% of bee species could not be assessed because of insufficient data [3]. In some cases, national Red Lists in Europe indicate that approximately up to 40% of bee species are threatened [9].

Probably the most important role of bees for humans is the pollination of wild plants with respect to the pollination of crops. Because most plant species are pollinated by insects, especially bees, the conservation of many habitats depends on their preservation. This is particularly important for wild species, many of which are pollen specialists on certain kinds of flowers, often with a strong preference for a given species. Hence, it is important to investigate wild bees in a certain region, in order to prevent their reduction due to the destruction of natural habitats or food competition with honey bees, especially when hive density is high.

In the Mediterranean Basin, Sardinia is the second largest island after Sicily, with a surface of 24,089 km². Sardinia shows unique flora and fauna characteristics related to paleogeographic connections with other Mediterranean cross-border regions [10]. It is located between 38°51′52″ and 41°15′42″ N latitude and between 8°8′ and 9°50′ E longitude, in the centre of the Western Mediterranean, neighbouring Corsica, about 300 km from the Ligurian and Provence coasts, 200 km from the Latium and Tunisian coasts, and 300 km from Sicily and the Balearic Islands [10,11]. The flora and fauna of the island are characterized by the presence of many peculiar species, with some endemic taxa shared with Corsica or other regions, including several species with a very restricted range. For example, the Sardinian Maghrebine, Sardinian Corsican, or Western Mediterranean areas have species such as *Anthophora nigrovittata* Dours, 1869, *Bombus ruderatus sardiniensis* Tournier, 1890, *B. terrestris sassaricus* Tournier, 1890, *Epeolus compar* Alfken, 1938, *Panurgus corsicus* Warncke, 1972, and *Tetraloniella dentata* amseli Alfken, 1938.

Several authors have studied Sardinian bees and other Hymenoptera Aculeata in the last century [12,13,14,15,16,17,18,19], and others have studied the ecological aspects of *Apis mellifera* L. and other pollinators [20,21]. To date, 316 species of bees are reported in Sardinia [22].

In the last decade, numerous studies on bees have been conducted in different islands of the Mediterranean Basin, such as Sicily [23,24,25,26,27,28], Malta [29,30], Cyprus [31], and the Balearic Islands [32]. However, the bee fauna of Sardinia still remains incomplete. Apart from some reports, such as *Lasioglossum zonulum* (Smith, 1848), *Sphecodes ferruginatus* von Hagens, 1882, and *Nomada corcyraea* Schmiedecknecht, 1882 [23,24], *Osmia spinulosa* (Kirby, 1802), and a newly described species, *Hoplitis occidentalis* Müller, 2012 [33,34], there is a lack of studies on the systematics, distribution, population trends, and ecology of bees in Sardinia [3,35]. It is very important to know and protect the potentially rich bee biodiversity of Sardinia before the different factors that now threaten wild pollinators in Europe could lead to its impairment.

This paper, in which 20 new records of bees are reported for Sardinia, following observations made in different coastal and mountain ecosystems, represents a first attempt to collect more information about bee biodiversity on the island.

## 2. Materials and Methods

**Figure 1:**
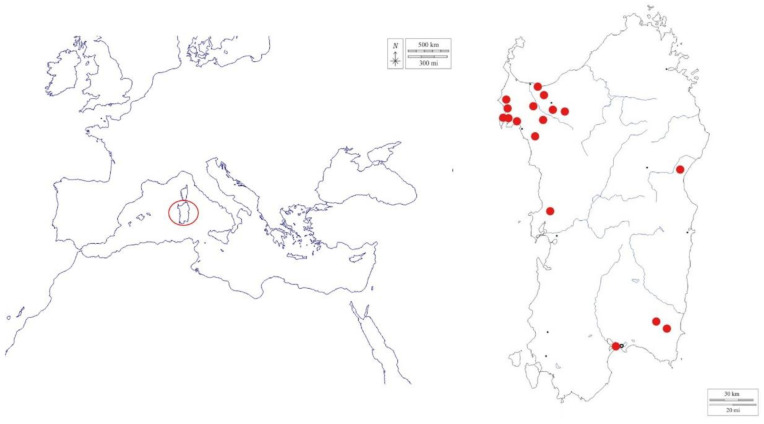
Position of Sardinia in the Mediterranean Basin and sampling sites (red dot) on the island.

**Figure 2:**
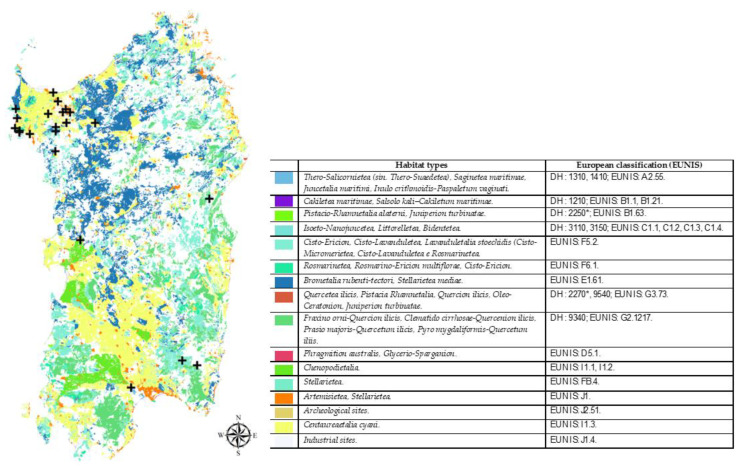
Mapping of habitats present in Sardinia [36,37] and sampling sites (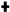) where the new Apoidea were recorded.

**Figure 3:**
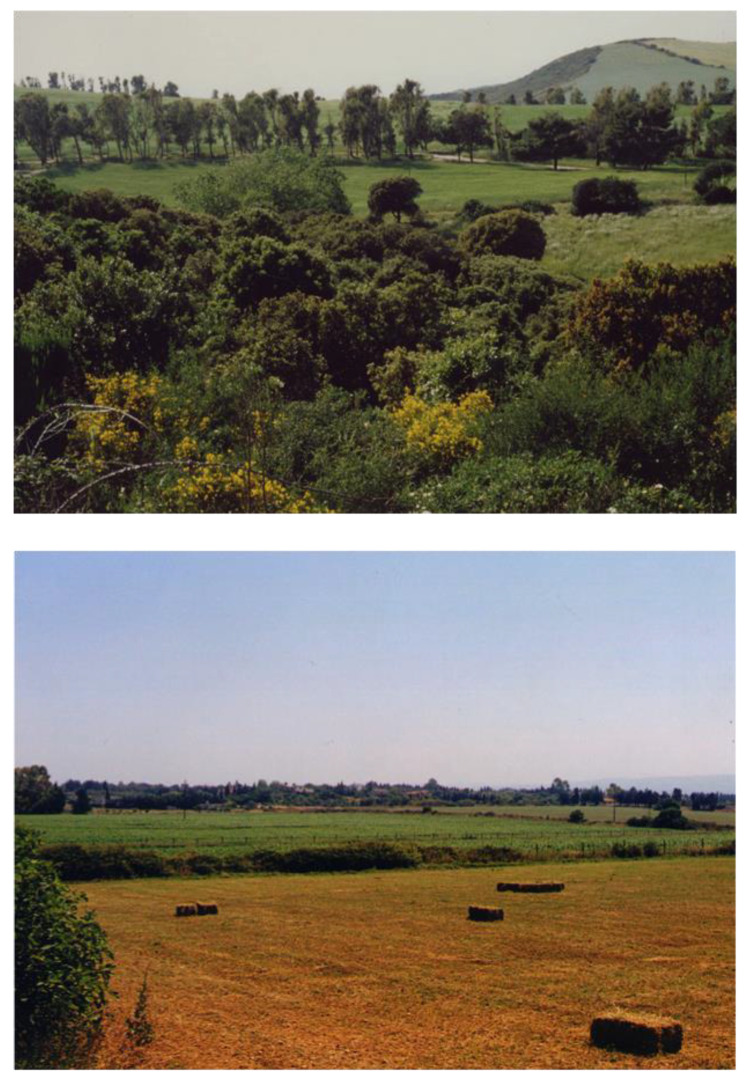
Two study habitats representative of northern Sardinia: a typical semi-natural farming area (Palmadula, SS; above) and a semi-intensive farming system (Ottava, SS; below).

Sardinia is included in the Mediterranean biogeographical region and is, therefore, only marginally composed of habitats characteristic of the continental region and, even less so, of the Alpine region. Some habitats of the island are indicated as priority in Natura 2000. Coordinates of the sampling sites as well as the location, mapping, and European classification of the habitats studied are shown in Figure 1 and Figure 2. The sampling sites represent various natural and anthropized habitats located in the main districts (i.e., Cagliari, Nuoro, Oristano, and Sassari) of the island. As an example, two sites representing a typical semi-natural area and a semi-intensive farming system of northern Sardinia, the area studied the most in this survey, are shown in Figure 3.

The surveys were conducted during the last decade, specifically by one of the authors (P.N.), in 20 natural sites, characterized by diverse altitudes, from sea level to 516 m a.s.l., and by different climatic and vegetational conditions, as follows:

### 2.1. Sampling Sites

#### 2.1.1. Cagliari District

*Assemini, Rio Flumini Mannu, 39.26, 9.00, 1 m a.s.l.*

*Burcei, Nord di Burcei, 39.403250, 9.349528, 430 m a.s.l.*

*Burcei, Rio Ollastu, 39.377278, 9.451333, 90 m a.s.l.*

#### 2.1.2. Nuoro District

*Dorgali, Oddoene, 40.257906, 9.541754, 150 m a.s.l.*

#### 2.1.3. Oristano District

*Milis, Santu Simoni, 40.04, 8.65, 70 m a.s.l.*

#### 2.1.4. Sassari District

*Alghero, Calich, Punta Dell’Eru, 40.599763, 8.295892, 3 m a.s.l.*

*Alghero, Mastr’Antoni, 40.608114, 8.222451, 25 m a.s.l.*

*Alghero, Cala Viola, 40.629641, 8.192253, 13 m a.s.l.*

*Alghero, Monte Doglia, 40.616917, 8.224944, 65 m a.s.l.*

*Olmedo, Strada Statale 291, Janna de Mare, 40.706944, 8.422417, 73 m a.s.l.*

*Ploaghe, 40.66, 8.75, 360 m a.s.l.*

*Porto Torres, Spiaggia di Platamona, 40.819722, 8.456758*, *1 m a.s.l.*

*Sassari, Ottava, 40.773194, 8.489889*, *70 m a.s.l.*

*Sassari, Palmadula, 40.731750, 8.196861*, *200 m a.s.l.*

*Sassari, Porto Ferro, 40.683111, 8.206528, 3 m a.s.l.*

*Sassari, in urbe, 225 m a.s.l.*

*Tissi, 40.66, 8.55, 200 m a.s.l.*

*Uri, Cuga, 40.615082, 8.475325, 110 m a.s.l.*

*Uri, Rio Carrabusu, 40.637330, 8.476420, 100 m a.s.l.*

*Villanova Monteleone, Sa Serra, 40.509423, 8.473922, 516 m a.s.l.*

### 2.2. Acronyms Used in the Text

AS—Alberto Satta, Sassari, Italy

CA—CREA-AA, Bologna, Italy

CM—Carlo Meloni, Cagliari, Italy

IF—Ignazio Floris, Sassari, Italy

MP—Michelina Pusceddu, Sassari, Italy

MQ—Marino Quaranta, Bologna, Italy

PN—Pietro Niolu, Alghero, Italy

RC—Roberto Catania, Catania, Italy

SB—Salvatore Bella, Catania, Italy

SF—Simone Flaminio, Bologna, Italy

US—University of Sassari, Italy

VN—Vittorio Nobile, Ragusa, Italy

The distribution, locality and date of collection, number of specimens, visited plants, and collector are given for each bee species. Bees were collected mainly on flowers using a hand net. Sampling was carried out between 9:00 and 17:00. As reported in the text, some specimens came from previous samplings carried out during the second part of the 21th century. All specimens were prepared dry and identified through the observation of sexual structures.

Specimen identification was supported by previous research studies [38,39,40,41,42,43,44,45,46,47,48,49]. Reference was made to the online checklist of the Western Palearctic Bees by Kuhlmann et al. [49], the Hymenoptera Atlas by Rasmont and Haubruge [50], and the Hymenoptera: Apoidea: Anthophila of Italy by Comba [22]. The classification used in this paper for supra-specific *taxa* followed Michener [2] and insect nomenclature was according to Polaszek [51]. The studied specimens are preserved in the collections of the authors and in the entomological collections of CA and US.

## 3. Results

**Figure 4:**
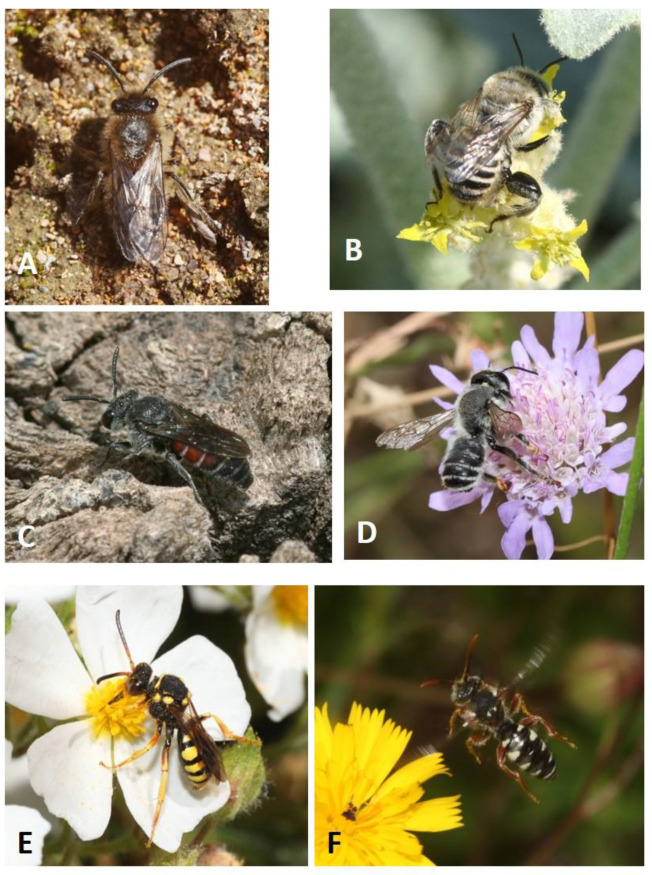
New species of pollinators (Hymenoptera: Apoidea) for Sardinia: (**A**) *Colletes cunicularius*; (**B**) *Lithurgus tibialis*; (**C**) *Dioxys cinctus*; (**D**) *Megachile ericetorum*; (**E**) *Nomada melathoracica*; and (**F**) *Nomada pulchra* (Photos: Pietro Niolu).

Overall, the following 20 species of Apoidea reported here belong to five different families and 11 genera (Table 1, Figure 4). Table 2 summarizes, comparatively, the data of the 20 species recorded in Italy and in other Mediterranean countries [52] before our study.

Fam. COLLETIDAE

Gen. *Colletes* Latreille, 1802
Colletes cunicularius (Linnaeus, 1761)
*Apis cunicularia* Linnaeus 1761. Linnaeus 1761: 422.

Examined specimens: Porto Ferro, 3.III.2019, 1 ♂, 19-23.II.2021, 9 ♂♂, PN leg. and det., US and RC coll.

Distribution: widespread in the Palaearctic region [49].

Range in Italy: this species is distributed throughout continental Italy.

*Colletes eous* Morice, 1904
*Colletes eous* Morice 1904. Morice 1904: 43–44.

Examined specimens: Cala Viola, 16.VI.2018, 1 ♂, PN leg. and det., US coll.

Distribution: widespread in Europe (except the colder areas), North Africa (Tunisia), and Southwestern Asia (Armenia, Azerbaijan, Georgia, and Turkey) [49].

Range in Italy: this species is distributed throughout continental Italy.

Fam. ANDRENIDAE

Gen. *Andrena* Fabricius, 1775
Andrena (Chlorandrena) humilis Imhoff, 1832
*Andrena humilis* Imhoff 1832. Isis (Oken) Jena: 1201.

Examined specimens: Burcei, Nord di Burcei, 17.IV.2019, 1 ♂, MQ leg., SF det., CA coll.; Burcei, Rio Ollastu, 18.IV.2019, 1 ♂, MQ leg., SF det., CA coll.

Distribution: widespread in the Palaearctic region and North Africa (Algeria, Morocco, and Tunisia) [49].

Range in Italy: this species is distributed throughout continental Italy and Sicily.

*Andrena (Euandrena) granulosa* Pérez, 1902
*Andrena granulosa* Pérez 1902. P.-v. Soc. linn. Bordeaux, 57: CLXXIX.

Examined specimens: Calich, Punta dell’Eru, 11.V.2019, 1 ♀, PN leg., MQ det., CA coll.

Distribution: widespread in Europe (except for the colder areas) and North Africa (Morocco) [47].

Range in Italy: this species is discontinuously present in the Italian peninsula.

*Andrena (Melandrena) cineraria* (Linnaeus, 1758)
*Apis cineraria* Linnaeus 1758. Syst. nat. (Ed. 10) 1: 575.

Examined specimens: Tissi, 15.V.1956, 1 ♂, VN det. and coll.

Distribution: widespread in the Palaearctic region [49].

Range in Italy: this species is distributed throughout continental Italy.

*Andrena (Micrandrena) tenuistriata* Pérez, 1895
*Andrena tenuistriata* Pérez, 1895. Pérez 1895: 44

Examined specimens: Olmedo, Strada Statale 291, Janna de Mare, 10.III.2019, 1 ♀, on *Calendula* sp., PN leg., SF det., CA coll.

Distribution: Southwestern Europe and North Africa (Morocco, Algeria, Tunisia, and Libia)

Range in Italy: this species is discontinuously present in the Italian peninsula and Sicily.

*Andrena (Micrandrena) rugulosa* Stöckhert, 1935
*Andrena rugulosa* Stöckhert, 1935 Dt. ent. Z. 1935: 66

Examined specimens: Burcei, Rio Ollastu, 18.IV.2019, 1 ♀, MQ leg., VN det., CA coll.

Distribution: Very rare species in Europe, with a distribution range from the northwestern part of the Alps to Crimea [47].

Range in Italy: so far known in Italy only in a locality in the extreme Italian border of the Trieste Karst [53].

*Andrena (Notandrena) pallitarsis* Pérez, 1903
*Andrena pallitarsis* Pérez 1903. P.-v. Soc. linn. Bordeaux, 58: LXXXIX.

Examined specimens: Villanova Monteleone, Sa Serra, 26.VIII.2017, 1 ♂, on *Foeniculum vulgare* Mill. (Apiaceae), PN leg., VN det., RC coll.

Distribution: widespread in Europe (including Urals), and West Asia (Armenia, and Georgia) [49].

Range in Italy: this species is present in the regions of northern Italy.

*Andrena (Suandrena) savignyi* Spinola, 1838
*Andrena savignyi* Spinola 1838. Annls Soc. ent. Fr., 7: 512.

Examined specimens: Platamona, 23.IX.2017, 4 ♀♀, MQ leg. and det., CA coll.

Distribution: Southwestern Europe, North Africa (Algeria, Egypt, Lybia, and Morocco) and West Asia (Israel and the United Arab Emirates) [49].

Range in Italy: this species is known in the regions of Central and Southern Italy.

Fam. HALICTIDAE

Gen. *Sphecodes* Latreille, 1804
*Sphecodes reticulatus* Thomson, 1870
*Sphecodes reticulatus* Thomson, 1870. Opuscula entomologica. Bd. 2. Håkan Ohlson, Lund, 98

Examined specimens: Dorgali, Oddoene, 9.VI.2018, 1 ♂, PN leg. and det.

Distribution: this species occurs throughout the Palearctic region but is rare within much of its distribution area.

Range in Italy: this species is distributed throughout continental Italy and Sicily.

Fam. MEGACHILIDAE

Gen. *Lithurgus* Latreille, 1825
*Lithurgus (Lithurgus) tibialis* Morawitz, 1875
*Lithurgus tibialis* Morawitz 1875. Fedtschenko, Izv. Imp. Obsestva. Lûbit. Estestv. Antropol. Etnogr., 19: 103.

Examined specimens: Alghero, Mastr’Antoni, 5.VIII.2018 1 ♂, on *Chrozophora tinctoria* (L.) A. Juss. (Euphorbiaceae); Uri, Cuga, 10.VII.2019, 1 ♂ PN leg. and det., CA and US coll.

Distribution: Southern Europe, Northern Africa, Eastern Mediterranean (Cyprus), Central Asia (Turkmenistan), and Southern Asia (Iran, and Pakistan) [31].

Range in Italy: this species is present only in Sicily.

Gen. *Chelostoma* Latreille, 1809
*Chelostoma (Chelostoma) emarginatum* (Nylander, 1856)
*Heriades emarginata* Nylander 1856. Mem. Soc. des Sciences Nat. de Cherbourg, 4: 109.

Examined specimens: Villanova Monteleone, Sa Serra, 7.V.2017, 1 ♀, on *Ranunculus* sp. (Ranunculaceae), PN leg., VN det., RC coll.

Distribution: widespread in Europe and Southwestern Asia (Iran and Turkey) [54].

Range in Italy: this species is distributed throughout continental Italy and Sicily.

Gen. *Dioxys* Lepeletier and Serville, 1825
*Dioxys cinctus* (Jurine, 1807)
*Trachusa cincta* Jurine 1807. Nouv. method. class. Hymenopter., 1: 253.

Examined specimens: Ploaghe, 16.V.1956, 1 ♀, VN det. and coll.

Distribution: Central Europe and North Africa [28].

Range in Italy: this species is distributed throughout continental Italy and Sicily.

Gen. *Coelioxys* Latreille, 1809
*Coelioxys (Allocoelioxys) caudatus* Spinola, 1838
*Coelioxys caudatus* Spinola 1838. Ann. Soc. ent. France, 7: 535.

Examined specimens: Milis, Santu Simoni, 9.VII.1974, 1 ♂, VN det. and coll.

Distribution: Southwestern Europe, North Africa (Algeria and Morocco), and Western Asia (Armenia, Azerbaijan, Georgia, Israel, and Turkey) [49].

Range in Italy: this species is discontinuously present in the Italian peninsula and in Sicily.

*Coelioxys (Allocoelioxys) obtusus* Pérez, 1884
*Coelioxys obtusus* Pérez 1884. Act. soc. Linn. Bordeaux, 37: 279.

Examined specimens: Rio Flumini Mannu, Assemini, 2.VII.1992, 4 ♂♂, on *Ammi visnaga* (L.) Lam. (Apiaceae), CM leg., VN det. and coll.

Distribution: Southwestern Europe, North Africa (Algeria, Egypt, and Morocco), and Western Asia (Armenia, Azerbaijan, Georgia, Iraq, and Turkey) [49].

Range in Italy: this species is discontinuously present in the Italian peninsula and in Sicily.

Gen. *Megachile* Latreille, 1802
*Megachile (Pseudomegachile) ericetorum* (Lepeletier, 1841)
*Megachile ericetorum* Lepeletier 1841. Hist. Nat. Insectes Hyménopt., 2: 341.

Examined specimens: Sassari, 18.VII.1997, 1 ♂, AS leg., MQ det., US coll.; Uri, Cuga, 25.VI.2019, 1 ♂, PN leg and det., US coll.

Distribution: widespread in Europe (except for the colder areas), Northern Africa, Eastern Mediterranean (Cyprus), Southern Asia (Iran), and Eastern Asia (China) [31].

Range in Italy: this species is distributed throughout continental Italy and Sicily.

Fam. APIDAE

Gen. *Nomada* Scopoli, 1770
*Nomada melathoracica* Imhoff, 1834
*Nomada melathoracica* Imhoff 1834. Isis 1834, p. 373.

Examined specimens: Porto Ferro, 28.IV.2018, 1♂, PN leg. and det., US coll.

Distribution: widespread in Europe (except for the colder areas) and Middle East (Turkey) [48].

Range in Italy: this species is known in the regions of Northern and Central Italy.

*Nomada pulchra* Arnold, 1888
*Nomada pulchra* Arnold, 1888. Horae Soc. ent. ross. 22, p. 202.

Examined specimens: Calich, Punta dell’Eru, 11.V.2019, 1 ♂, PN leg., RC det., CA coll.

Distribution: widespread in Europe, Middle East (Turkey), and Asia (Kazakhstan and Eastern Siberia) [48].

New for Italy and Sardinia.

Gen. *Eucera* Scopoli, 1770
*Eucera (Eucera) proxima* Morawitz, 1875
*Eucera proxima* Morawitz 1875. Fedtschenk, Reise Turkestan, Apidae I., p.61.

Examined specimens: Uri, Rio Carrabusu, 19.IV.2018, 1 ♀, PN leg., MQ det., CA coll.

Distribution: widespread in Europe (except the colder areas), Eastern Mediterranean (Cyprus) Western Asia (Syria and Turkey), Central Asia (Turkmenistan, Uzbekistan, and Tajikistan), and Southern Asia (Iran) [31].

Range in Italy: this species is discontinuously present in the Italian peninsula and in Sicily.

Gen. *Tetralonia* Spinola, 1838
*Tetralonia (Tetralonia) malvae* (Rossi, 1790)
*Apis malvae* Rossi 1790. Fauna Etrusca II., p. 107.

Examined specimens: Dorgali, Oddoene, 10.VI.2018, 1 ♀, 1 ♂, on *Althaea rosea* L. (Malvaceae), PN leg. and det., US coll.

Distribution: widespread in Europe, Eastern Mediterranean (Cyprus), and Western Asia (Turkey) [31].

Range in Italy: this species is discontinuously present in the Italian peninsula and in Sicily.

Shortly before submitting our paper, we became aware of the existence of a record for this species: Gonnosfanadiga, 39.49361, 8.66, (sex?), 6.VI.1943, D. Dale leg., B. Pittioni det., preserved at the Snow Entomological Museum, University of Kansas (Kansas, USA) [55].

Our specimens confirm the presence of the species on the island 78 years later.

## 4. Discussion

Currently, 316 species of bees are known in Sardinia [22]. Data on bees are lacking for most of the central and southern areas of the island, whereas much more data are available for the northwestern area of Sardinia. This was thanks to the work carried out by the Department of Agricultural Sciences of the University of Sassari, especially from 1997 to 2010, when a survey on wild pollinators was conducted, jointly with eight other research teams in Italy, using a unique scheme for monitoring the bee fauna [18]. The increased knowledge of this group of insects is of primary importance for knowing the local populations of pollinators of wild and cultivated plants [18,56]. In this context, studies have focused on the role of pollinators in certain agroecosystems or crops, such as the pollinators of white clover (*Trifolium repens* L.) in northern Sardinia [57], where the primary role of the wild bees belonging to *Andrena*, *Megachile*, and *Osmia* genera was evidenced. A study [58] on the pollinating insects of *Hedysarum coronarium* L. in northern Sardinia reported a total of 18 wild bee species, among which *Eucera numida* Lep. visited the crop most actively, followed by the endemic *Bombus* subspecies (*B. ruderatus sardiniensis* and *B. terrestris sassaricus*), *Osmia bicornis* L., and *Megachile parietina* Geoffroy. A survey of bee fauna in two different agroecosystems (semi-intensive vs. extensive) located in northern Sardinia (Nurra) [21] evidenced 28 species belonging to the Andrenidae, Halictidae, Megachilidae, and Apidae families in the semi-intensive agroecosystem compared to 31 species in the extensive agroecosystem, which showed the same families plus Colletidae. The results indicate that agriculture intensification did not lead to a significant decrease in species richness. However, the Sorensen’s community index showed a medium similarity between the insect composition of the two landscapes. The effect of agricultural intensification was particularly evident on Collectidae, Andrenidae, and Halictidae species that usually use the soil as nesting sites. In fact, these families were more represented in the extensive site, probably thanks to the minimum tillage and the wider availability of uncultivated zones compared to the semi-intensive area. On the contrary, the semi-intensive landscape had a higher number of Megachilidae and Apidae species recorded, compared to the extensive one, probably because of the presence of forage legume crops.

## 5. Conclusions

Here, we report the following 11 genera of Hymenoptera Apoidea for Sardinia: *Colletes* (two species), *Andrena* (seven spp.), *Sphecodes* (one sp.), *Lithurgus* (one sp.), *Chelostoma* (one sp.), *Dioxys* (one sp.), *Coelioxys* (two spp.), *Megachile* (one sp.), *Nomada* (two spp.), *Eucera* (one sp.), and *Tetralonia* (one sp.). Precisely, the following 20 new species are reported as first records for the island of Sardinia: *Colletes cunicularius*, *C. eous* (Colletidae), *Andrena humilis*, *A. granulosa*, *A. cineraria*, *A. tenuistriata*, *A. rugulosa*, *A. pallitarsis*, *A. savignyi* (Andrenidae), *Sphecodes reticulatus* (Halictidae), *Lithurgus tibialis*, *Chelostoma emarginatum*, *Dioxys cinctus*, *Coelioxys caudatus*, *C. obtusus*, *Megachile ericetorum* (Megachilidae), *Nomada melathoracica*, *N. pulchra*, *Eucera proxima*, and *Tetralonia malvae* (Apidae). Moreover, *N. pulchra* is new not only for Sardinia but also for Italy.

Understanding the diversity of pollinators in an insular context and how it can be preserved is an important step for better understanding the biodiversity of this fauna at the global level. In fact, the latter certainly requires more efforts to collect observational and experimental data, to monitor the situation, and to conduct detailed surveys to determine pollinator diversity and the threats behind their decline. In particular, in the islands, the pollinator diversity is the result of a long and diffuse coevolution with floral diversity and nest conditions, which lead, thanks to isolation, to speciation processes with richness of endemism.

## Figures and Tables

**Table 1:** List of recorded species of Apoidea that were new for Sardinia (Sa) and, in one case, for Italy (IT).

N°	Taxon	N° of Specimens	Botanical Choice	New for Sa/IT
**Fam. Colletidae**				
**1**	*Colletes cunicularius* (Linnaeus, 1761)	10		Sa
**2**	*Colletes eous* Morice, 1904	1		Sa
**Fam. Andrenidae**				
**3**	*Andrena* (*Chlorandrena*) *humilis* Imhoff, 1832	2		Sa
**4**	*Andrena* (*Euandrena*) *granulosa* Pérez, 1902	1		Sa
**5**	*Andrena* (*Melandrena*) *cineraria* (Linnaeus, 1758)	1		Sa
**6**	*Andrena* (*Micrandrena*) *tenuistriata* Pérez, 1895	1	Asteraceae	Sa
**7**	*Andrena* (*Micrandrena*) *rugulosa* Stöckhert, 1935	1		Sa
**8**	*Andrena* (*Notandrena*) *pallitarsis* Pérez, 1903	1	Apiaceae	Sa
**9**	*Andrena* (*Suandrena*) *savignyi* Spinola, 1838	4		Sa
**Fam. Halictidae**				
**10**	*Sphecodes reticulatus* Thomson, 1870	1		Sa
**Fam. Megachilidae**				
**11**	*Lithurgus* (*Lithurgus*) *tibialis* Morawitz, 1875	2	Euphorbiaceae	Sa
**12**	*Chelostoma* (*Chelostoma*) *emarginatum* (Nylander, 1856)	1	Ranunculaceae	Sa
**13**	*Dioxys cinctus* (Jurine, 1807)	1		Sa
**14**	*Coelioxys* (*Allocoelioxys*) *caudatus* Spinola, 1838	1		Sa
**15**	*Coelioxys* (*Allocoelioxys*) *obtusus* Pérez, 1884	4	Apiaceae	Sa
**16**	*Megachile* (*Pseudomegachile*) *ericetorum* (Lepeletier, 1841)	2		Sa
**Fam. Apidae**				
**17**	*Nomada melathoracica* Imhoff, 1834	1		Sa
**18**	*Nomada pulchra* Arnold, 1888	1		IT, Sa
**19**	*Eucera* (*Eucera*) *proxima* Morawitz, 1875	1		Sa
**20**	*Tetralonia* (*Tetralonia*) *malvae* (Rossi, 1790)	2	Malvaceae	Sa

**Table 2:** Comparison between data on the presence (x) of the twenty species of Apoidea in Italy and in Mediterranean coutries.

Genus	Species	Italy	France	Spain	Morocco	Tunisia	Algeria
*Andrena*	*Andrena cineraria*	x	x	x			
*Andrena*	*Andrena granulosa*	x	x	x	x		
*Andrena*	*Andrena humilis*	x	x	x	x	x	x
*Andrena*	*Andrena pallitarsis*	x	x	x			
*Andrena*	*Andrena rugulosa*	x	x				
*Andrena*	*Andrena savignyi*	x	x	x	x	x	x
*Andrena*	*Andrena tenuistriata*	x	x	x	x	x	x
*Chelostoma*	*Chelostoma emarginatum*	x	x	x		x	x
*Coelioxys*	*Coelioxys caudata*	x	x	x	x		x
*Coelioxys*	*Coelioxys obtusa*	x	x	x	x	x	x
*Colletes*	*Colletes cunicularius*	x	x	x			
*Colletes*	*Colletes eous*	x		x		x	
*Dioxys*	*Dioxys cincta*	x	x	x	x	x	x
*Eucera*	*Eucera proxima*	x	x	x			
*Lithurgus*	*Lithurgus tibialis*	x		x			
*Megachile*	*Megachile ericetorum*	x	x	x	x	x	x
*Nomada*	*Nomada melathoracica*	x	x	x			
*Nomada*	*Nomada pulchra*		x				
*Sphecodes*	*Sphecodes reticulatus*	x	x	x			
*Tetralonia*	*Tetralonia malvae*	x	x	x			

## Data Availability

The data presented in this study are available on request from the corresponding author.
